# Using a Brief Mental Imagery Competing Task to Reduce the Number of Intrusive Memories: Exploratory Case Series With Trauma-Exposed Women

**DOI:** 10.2196/37382

**Published:** 2022-07-20

**Authors:** Kristjana Thorarinsdottir, Emily A Holmes, Johann Hardarson, Elin S Stephenssen, Marianna H Jonasdottir, Marie Kanstrup, Laura Singh, Arna Hauksdottir, Thorhildur Halldorsdottir, Berglind Gudmundsdottir, Edda Thordardottir, Unnur Valdimarsdottir, Andri Bjornsson

**Affiliations:** 1 Department of Psychology University of Iceland Reykjavík Iceland; 2 Department of Psychology Uppsala University Uppsala Sweden; 3 Swedish Collegium for Advanced Study Uppsala University Uppsala Sweden; 4 The Center of Public Health Sciences University of Iceland Reykjavik Iceland; 5 Department of Psychology University of Reykjavik Reykjavik Iceland; 6 Faculty of Medicine University of Iceland Reykjavik Iceland; 7 The National University Hospital of Iceland University of Iceland Reykjavik Iceland; 8 Department of Epidemiology Harvard TH Chan School of Public Health Boston, MA United States

**Keywords:** trauma, intrusive memories, visuospatial task, Tetris gameplay, mental imagery, imagery competing task, case series, mobile phone, posttraumatic stress

## Abstract

**Background:**

Novel interventions should be developed for people who have undergone psychological trauma. In a previous case study, we found that the number of intrusive memories of trauma could be reduced with a novel intervention. The intervention included a brief memory reminder, a visuospatial task and mental rotation, and targeted trauma memory hotspots one at a time in separate sessions.

**Objective:**

This case series (N=3) extended the first case study with 3 new cases to determine whether a similar pattern of beneficial results is observed. We explored whether the brief intervention would result in reduced numbers of intrusive memories and whether it would impact symptoms of posttraumatic stress, depression and anxiety, and general functioning. Acceptability of the intervention was also explored.

**Methods:**

A total of 3 women completed the study: 2 with posttraumatic stress disorder and other comorbidities and 1 with subthreshold posttraumatic stress disorder. The primary outcome was the change in the number of intrusive memories from the baseline phase to the intervention phase and at the 1-month follow-up, with an assessment of the intrusion frequency at 3 months. Participants monitored the number of intrusive memories in a daily diary for 1 week at baseline, for maximum of 6 weeks during the intervention phase and for 1 week at the 1-month and 3-month follow-ups. The intervention was delivered in person or digitally, with guidance from a clinical psychologist. A repeated AB design was used (*A* was a preintervention baseline phase and *B* intervention phase). Intrusions were targeted individually, creating repetitions of an AB design.

**Results:**

The total number of intrusive memories was reduced from the baseline to the intervention phase for all participants. The total number for participant 3 (P3) reduced from 38.8 per week during the baseline phase to 18.0 per week in the intervention phase. It was 13 at the 3-month follow-up. The total number for P4 reduced from 10.8 per week at baseline to 4.7 per week in the intervention phase. It was 0 at the 3-month follow-up. The total number for P5 was reduced from 33.7 at baseline to 20.7 per week in the intervention phase. It was 8 at the 3-month follow-up. All participants reported reduction in posttraumatic stress symptoms in the postintervention phase. Depression and anxiety symptoms reduced in 2 of the 3 participants in the postintervention phase. Acceptability was favorable.

**Conclusions:**

We observed good compliance with the intervention and intrusive memory diary in all 3 cases. The number of intrusive memories was reduced for all participants during the intervention phase and at the 1-month follow-up, with some improvement in other symptoms and functioning. Further research should explore the remote delivery of the intervention and whether nonspecialists can deliver the intervention effectively.

## Introduction

### Background

Most people experience psychological trauma (eg, accidents or interpersonal violence) in their lives [1,2], and many (up to 37%) develop posttraumatic stress disorder (PTSD) after such experiences [3]. Intrusive memories are the core clinical symptoms of PTSD and are within the intrusion symptoms criterion of PTSD in the Diagnostic and Statistical Manual of Mental Disorders, Fifth Edition (DSM-5) [1,4]. Intrusive memories are persistent, unwanted upsetting memories of the traumatic event [1]. In their most extreme form, they can include reliving the traumatic event as if it were happening again (flashbacks). Other intrusion symptoms include dreams or nightmares about the traumatic event and emotional distress or physical reactivity after exposure to reminders of the traumatic event. Other symptom criteria include avoidance of memories or reminders of the trauma, along with negative alterations in cognition and mood [1]. Posttraumatic stress symptoms, even when subthreshold for a diagnosis of PTSD, can be associated with substantial distress, functional impairment, and comorbidity [5].

As noted previously [6], although evidence-based treatments for PTSD exist [7,8], the existing treatment options have some limitations. For example, current treatments require trauma survivors to talk in detail about the traumatic experience, which can be distressing, and many are reluctant to discuss their trauma in depth [9]. Dropout rates during PTSD treatment are high, up to 48% in clinical trials and approximately 18% overall and may be higher outside research trial settings in clinical practice [10-12]. Finally, existing options are time consuming, typically requiring numerous sessions, and there is often a lack of treatment providers specializing in empirically validated treatment of PTSD. Similar to numerous other countries, Iceland has mental health services that lack the capacity to offer treatments when needed by trauma survivors at the scale needed. These limitations to current treatments make the search for additional scalable treatment alternatives imperative.

A novel brief and simple intervention to reduce the number of intrusive memories after trauma has been developed based on cognitive science, as described elsewhere [13,14]. This intervention takes a single-symptom approach (not an entire disorder). The intervention includes a *brief memory reminder* cue for one *specific* intrusive memory of trauma, followed by a 25-minute Tetris gameplay with mental rotation (ie, actively rotating the blocks in one’s mind eye to best make lines; [15]). The intervention was initially examined based on recent memories of trauma [16-18]. It has been further explored for *older memories* of trauma using case studies and case series approaches [6,19-21]. These studies involved in-person delivery, that is, face-to-face sessions guided by a clinical psychologist or researcher.

We adapted the intervention for women in Iceland who experienced intrusive memories of trauma, as reported in a recent case study [6]. Some of the details of this case are now summarized for context and comparison with the 3 new cases presented here. As previously reported, the participant was a woman in her fifties with 4 distinct intrusive memories from a traumatic event that happened in childhood, that is, the intrusive memories were decades old. Each specific memory was targeted in a session (in person) with a clinical psychologist with expertise in trauma. The memory reminder used was to *briefly* bring the visual content of the memory to mind without becoming emotionally overwhelmed by a method agreed with the participant (here, for example, choosing 1 of her 4 specific memories to be targeted using the diary, then thinking about the memory for a few seconds only, and letting the psychologist know when the memory was in their mind). Next, the participant was taught to use mental rotation. She then played Tetris for 25 minutes using mental rotation. She monitored her specific intrusive memories in a daily diary so that the impact of the intervention on a distinct intrusive memory could be easily observed. The total number of intrusive memories decreased from 12.6 per week at baseline to 6.1 per week in the postintervention phase. Furthermore, the number of intrusive memories continued to reduce to only 1 memory per week at the 3-month follow-up. Symptoms of posttraumatic stress and depression and anxiety reduced in the postintervention phase, whereas functioning improved. The participants considered the intervention to be an acceptable way to reduce the number of intrusive memories. The next step in exploring the effects of the intervention involves examining if they extend to other cases of women after trauma and whether remote (rather than in person) delivery is a feasible delivery method given restrictions occurring during the COVID-19 pandemic (eg, isolation) [22].

### Objectives

In this case series, we aimed to extend our previous case study to a short case series of trauma-exposed women in Iceland, drawn from an epidemiological study of trauma experienced by women in Iceland. The intervention sessions took place either in person in a university setting or remotely using a web-based platform. We examined whether the novel intervention approach could reduce the number of intrusive memories of trauma (primary outcome) and whether reductions were maintained at follow-up (1 and 3 months). As before, the brief intervention was guided by a clinical psychologist, targeting one distinct intrusive memory at a time per session. The acceptability of the intervention was also explored along with adaptions in intervention delivery. Again, we explored whether having fewer intrusive traumatic memories would also be associated with improvements in general functioning, posttraumatic stress, and depression and anxiety symptoms (secondary outcomes). The design adopted here can be described as a within-subject *repeated* AB design in which each specific memory is targeted in separate sessions, so that we can consider the effects of an individual intervention session on each specific memory over time [6,20].

As in our previous study [6], we predicted that participants would report fewer intrusive memories (primary outcome) during the intervention phase than in the preceding baseline phase and that the reduction in the number of intrusions would be maintained at the 1-month follow-up in the diary. In addition, we explored a 3-month follow-up using a diary. We expected that the number of targeted intrusive memories would decrease relative to that of nontargeted memories. We also examined whether having fewer intrusive memories would be associated with reductions in symptoms of posttraumatic stress and depression and anxiety and associated with improvements in general functioning (secondary outcomes). Furthermore, we explored the feasibility and acceptability of the intervention, alongside adaptions in intervention delivery format, that is, remote (web-based) delivery.

## Methods

### Participants

Participants were drawn from an epidemiological study of trauma experienced by women in Iceland (as in our previous case study [6]). As described previously [6], women who participated in a substudy of the Stress-And-Gene-Analysis (SAGA) cohort study were screened for eligibility. The SAGA cohort study was a population-based longitudinal cohort study of women in Iceland who completed an extensive questionnaire on trauma history and mental health (baseline data collection was completed on July 1, 2019). The Social Trauma Project substudy compared 2 samples from the SAGA cohort study, with a probable diagnosis of PTSD (ie, Posttraumatic Stress Disorder Checklist-5 [PCL-5] score of ≥33; see *Measures* section) or not likely PTSD (ie, PCL-5 score in the lowest fifth), using clinical interviews. In all, 2 semistructured interviews were administered in the substudy (ie, the Mini International Neuropsychiatric Interview [MINI], which was also used to assess the exclusion criteria for this study, and the Clinician-Administered PTSD Scale for DSM-5 (CAPS-5; see *Measures* section).

Women who took part in the Social Trauma Project substudy (both likely PTSD group and not likely PTSD group) were screened for the presence of intrusive memories of trauma, as in our previous case study [6]. As before, screening included a short description of intrusive memories (memories that include sensory impressions such as sight, sound, and so on; often pictures or a film clip that pops into the mind’s eye; are distressing and occur involuntarily). Next, they were asked questions regarding the presence of this symptom to assess their eligibility for participation in this study (“Do you have intrusive memories of trauma? If yes, how often in the past week have you experienced such memories?” and “How often have you experienced intrusive memories a week in the past four weeks?”).

**Figure 1:**
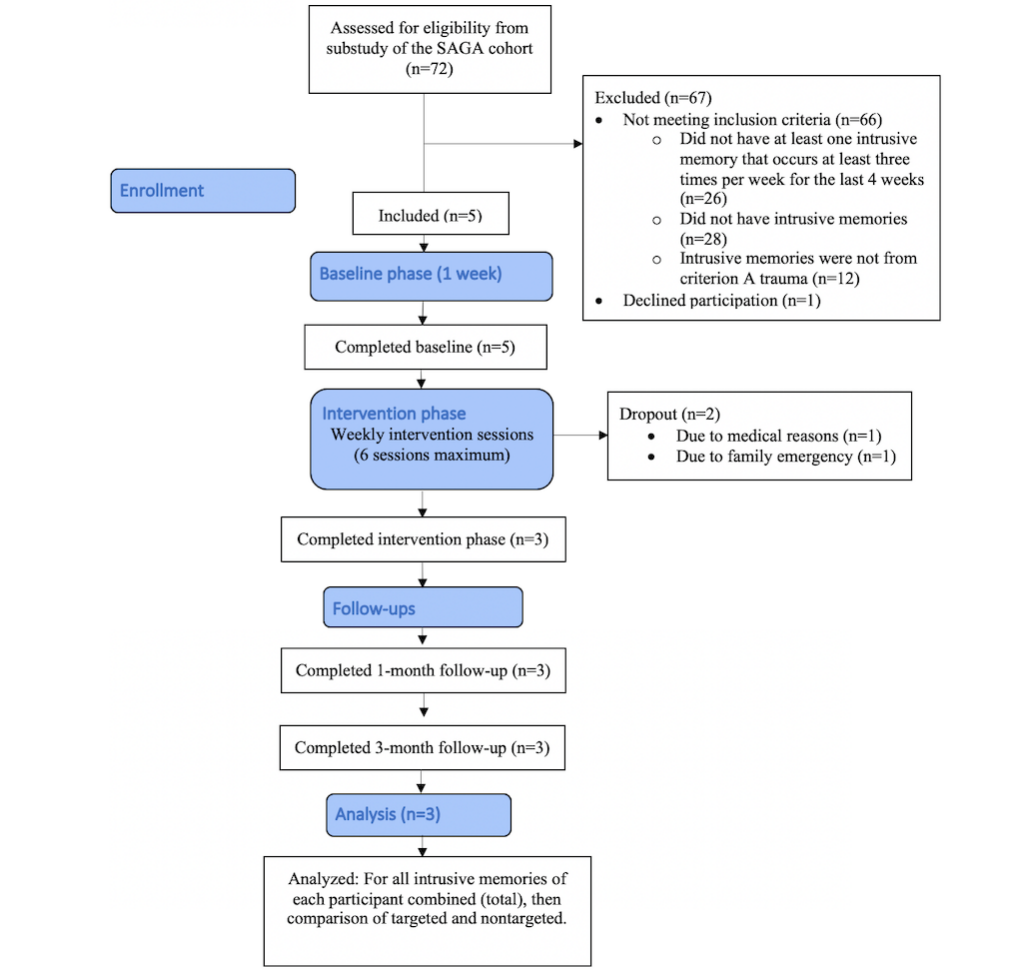
Adapted CONSORT (Consolidated Standards of Reporting Trials) flow diagram for the study. SAGA: Stress-And-Gene-Analysis.

In this study, 72 women from the substudy, who provided their consent to be contacted for further research were assessed for inclusion in this study (Figure 1).

Inclusion criteria were (1) having experienced criterion A trauma as defined by DSM-5 [1] (using criterion A on CAPS-5), (2) having at least one intrusive memory that occurs at least three times per week for the last 4 weeks (How many intrusive memories have you experienced in the last 4 weeks on average?), (3) being able and willing to attend 3 to 8 sessions with the researcher, (4) being able and willing to monitor intrusive memories in daily life, (5) having access to a smartphone, and (f) being able to speak and read study materials in Icelandic. The exclusion criteria were (1) current psychotic disorder (assessed with the MINI), (b) current manic episode (assessed with MINI), and (c) being acutely suicidal (assessed with the MINI).

In all, 5 women who met the inclusion criteria were included, ranging in age from 39 to 66 years (mean 49, SD 11 years). Participants are referred to as P1, P2, P3, P4, and P5. The initial 2 participants recruited did not complete the intervention sessions due to nonsuitability of the study timing in their lives (medical and family issues). Both P1 and P2 were excluded from the data analyses, although data from the baseline were collected. After P1 and P2 wished to cease their participation, a question was added to the recruitment process asking if the participants foresaw any obstacles to their participation in the study. P1 did not meet any diagnostic criteria for psychological disorders according to the MINI. P2 met the diagnostic criteria for bipolar disorder and reported subthreshold PTSD symptoms.

A total of 3 patients completed the study, 2 (67%) with PTSD and other comorbidities and 1 (33%) with subthreshold PTSD. P3, woman aged ≥40 years, met the criteria for major depressive disorder and PTSD. P4, a woman aged ≥60 years, did not meet any diagnostic criteria but reported subthreshold PTSD according to the CAPS-5. P5, a woman aged ≥50 years, met the diagnostic criteria for major depressive disorder, social anxiety disorder, and PTSD. P3 reported having 10 different intrusive memories of physical violence that occurred in childhood. P4 reported having 3 different intrusive memories from childhood sexual abuse, and P5 reported 6 different intrusive memories from childhood sexual abuse.

### Design

The case series used a single-symptom approach, in which each intrusive memory was targeted in different sessions, that is, one at a time [6,19]. Specifically, participants distinguished the content of their different intrusive memories and described them briefly; for example, for a participant who had four distinct intrusions, they may label them as (1) broken glass, (2) man’s face, (3) blood on door, and (4) red car (the examples used are fictitious to preserve anonymity). Participants then monitored the occurrence of each distinct intrusion over time.

Thus, the design for each participant was a *repeated AB design* whereby the length of baseline (*A*, before intervention, monitoring only) and intervention (*B*) phases varied for each distinct intrusion, according to which intervention session the intrusive memory was targeted in. Thus, the baseline phases for each distinct intrusive memory were used as control periods in the comparison with the intervention, that is, the number before and number after being targeted.

A daily diary was used to monitor the number of each intrusive memory over time. That is, for 1 week before intervention, then over 6 weeks (maximum) of intervention; then for 1 week at the 1- and 3-month follow-ups. Intrusive memories were targeted individually in up to 6 intervention sessions. These sessions were guided by a clinical psychologist, who was a specialist in trauma-focused cognitive behavior therapy. After the first session, participants were able to self-administer the intervention if they wished for memories that they had already targeted in the session. P3 received 2 repetitions of an AB design, P4 only targeted 1 memory (and thus no repetitions of an AB design), and P5 received 4 repetitions of an AB design.

The primary outcome was the change in the number of intrusive memories per week from baseline to the intervention phase and to the long-term follow-ups (1 and 3 months). Participants further completed secondary outcomes—self-report measures for posttraumatic stress, depression and anxiety symptoms, and functional impairment—at baseline, the last intervention session, and 1- and 3-month follow-ups.

### Procedure

#### Training

Researchers delivering the intervention (KT and JH; both licensed clinical psychologists trained in trauma-focused cognitive behavior therapy) underwent training and received clinical supervision, promoting adequate intervention delivery and protocol adherence. Training involved 2 in vivo workshops for 3 days and then again approximately 6 months later for 2 days, delivered by psychologists with expertise in developing the intervention and delivering it in other settings (MK and EAH). The theoretical background and practical aspects of the intervention (eg, how to explain and use the primary outcome measure) were covered in the workshops, as well as role-playing with trainers and feedback until sufficient performance was reached. While data collection was ongoing, the researchers received supervision. Such supervision included weekly supervision meetings, as well as more *in real time* support from a clinical supervisor via telephone directly after participant sessions regarding any case specific adaptions needed (EAH, MK, and AB). Twice a month, the researchers joined remote (Zoom; Zoom Video Communications) peer-group training meetings with other international researchers about the intervention (convened by EAH and LS).

#### Baseline Session

A similar procedure was followed as in our previous case study [6]; in the baseline session, the researcher explained the nature of intrusive memories (ie, memories that include sensory impressions such as sight, sound, and so on; are predominantly similar to pictures or a film clip in the mind’s eye; and are distressing and occur involuntarily). Participants identified their intrusive memories by giving a brief verbal account of the intrusion’s visual content using only a few words. Researchers noted down the image’s description on a *hotspots* sheet in a way that the participant could also see it. Participants then labeled each intrusive memory with a symbol (the first memory was labeled *A*, the second memory *B*, etc) and were instructed on how to monitor their frequency each day in a pen-and-paper diary (primary outcome measure). To indicate when a certain memory was experienced, the participants recorded the symbol corresponding to a given memory for each time frame of that day. Each diary was divided into 7 days and each day, into 4 periods (see *Measures* section). The participants also completed baseline questionnaires (secondary outcomes) in the baseline session.

#### Intervention Sessions

In each intervention session (maximum 6 sessions), the participant chose 1 intrusive memory to target (by looking through their diary entries) and completed the intervention procedure (guided by the researcher). The memory chosen could be the most distressing or frequent or one chosen to be targeted by the participant for other reasons. As in our previous case study [6], the intervention consisted of a brief memory reminder, that is, briefly thinking about the intrusive memory to bring the image to mind without it becoming emotionally overwhelming. Please note that the approach to bringing the memory to mind here differs procedurally from the memory reminder method in the studies by Kessler et al [19] or Kanstrup et al [20]. Participants were told, “To make the game as useful as possible, we first had to make sure the memory was in your mind before using the intervention. So, I want to ask you what do you think would be the best way for you to bring this memory to mind without it becoming emotionally overwhelming?” They then discussed with the researcher options for the best way for them to bring the memory into mind without it emotionally becoming overwhelming. To do this, they were given examples of writing it down briefly and not discussing it with the psychologist, thinking about it briefly again and not talking about it in detail, or finding another method. Here, all participants chose to bring to mind the memory they had chosen to target by briefly thinking about it for a few seconds with their eyes open and telling the psychologist when it had come fully to mind (without talking about it in detail). The psychologist confirmed that the participants were able to picture their memory (ie, see it in their mind’s eye) before moving to the instructions about the gameplay.

After the memory reminder procedure, participants were trained on how to use the Tetris game and practiced using mental rotation. They then played Tetris using mental rotation for 25 minutes [15]. For in-person meetings, the Tetris gameplay was delivered with a Nintendo DS 10.1-inch screen, set to *Marathon* mode with the ghost piece off. When an intervention session took place remotely with a video call, Tetris gameplay was performed on the participants’ own computer with a shared screen so that the researcher could monitor the gameplay, especially regarding mental rotation. Only one distinct intrusion was targeted for each session. To select which intrusion was targeted, the participant (not the researchers) selected which intrusive memory it was. At the end of the last intervention session, secondary outcome measures were completed again.

Participants were invited to self-administer the intervention for memories already targeted in a session using a mobile version of the Tetris created by Electronic Arts [23]. For example, when the intrusion came to mind involuntarily in daily life, they were told to use a similar procedure as they had learned with the researcher in session.

When the COVID-19 pandemic started (the University of Iceland closed on March 19, 2020), researchers switched to remote (rather than in person) delivery through Kara Connect, which is a General Data Protection Regulation–compliant web-based platform certified by the Icelandic Directorate of Health. The last intervention session for P3 was performed remotely, intervention sessions 2 and onward were performed remotely for P4, and all sessions were remotely delivered for P5. Tetris was played on the web on the participants’ computers (ghost piece off and sound set to 0%) with a shared screen so that the researcher could monitor participants’ gameplay via Kara Connect, which increased the likelihood of instruction adherence, especially regarding the use of mental rotation.

#### Follow-up

At both the 1- and 3-month follow-up time points, participants recorded the number of intrusions in their diary daily again for 1 week and completed the secondary outcome measures. Data were entered via laptop into a REDCap (Research Electronic Data Capture; Vanderbilt University) database (REDCap), an encrypted electronic software stored on a secure server [24].

### Measures

#### Eligibility Assessments (Part of the SAGA Cohort Substudy)

Please note that the measures described here have already been described in our previous case study [6] and are repeated here for clarity.

*The Clinician-Administered PTSD Scale (CAPS-5)* is a 30-item semistructured interview used to assess symptoms of PTSD from an index of trauma and symptom severity (in the past month) according to DSM-5 [1]. Items are scored on a 5-point Likert scale (0=mild or subthreshold; 4=extreme or incapacitating). A symptom rating of 2 (ie, moderate) was the threshold for a possible diagnosis. For each symptom, frequency and intensity were assessed and rated separately. The CAPS-5 has excellent internal consistency (Cronbach *α*=.88), test-retest reliability (*α*=.83), and good convergent validity (*α*=.83) [25]

The MINI for the Diagnostic and Statistical Manual of Mental Disorders, Fourth Edition, assesses Axis I psychiatric disorders according to the Diagnostic and Statistical Manual of Mental Disorders, Fourth Edition, using a structured diagnostic interview. For most diagnoses, the MINI has good sensitivity and specificity [25]. Interrater and test-retest reliability is good, with κs in the high to very high range (κs=0.79-1.00) [26].

#### Primary Outcome Measure

*The intrusive memory diary*, a pen-and-paper diary similar to that used in Thorarinsdottir et al [6], was adapted from previous experimental and clinical studies [16,20]. It involves daily recording, for 4 time points each day (morning, afternoon, evening, and night) for 1 week. The diary instructions defined the nature of intrusive memories as distressing and involuntary mental images (such as visual images or a film in the mind’s eye). Participants were asked not to record voluntary (ie, deliberately recalled) thoughts or involuntary verbal thoughts without sensory content (intrusive verbal thoughts that had an imagery component could be included). Participants monitored the occurrence (or nonoccurrence) of their intrusive memories in the daily diary for 1 week before the intervention (baseline phase), for a maximum of 6 weeks during the intervention phase and then again for 1 week at both the 1- and 3-month follow-ups (note this was a daily diary, see retrospective amendment to clinical trial registration [CTR] NCT04209283). When indicating having an intrusion, participants used the symbol corresponding to that specific memory (eg, A or B as noted earlier), and therefore, it was possible to examine change in frequency (here calculated as the number per week) for each distinct memory individually.

The primary outcome was the change in the number of intrusive memories from the baseline to the intervention phase and at the 1-month follow-up. The original CTR additionally prespecified a measure of intrusive memories at the 3-month follow-up (“Change in the number of intrusive memories of trauma from baseline to 3 month follow-up”) but incorrectly stated that the measure was “Questions about the frequency of intrusive memories for the past day or week (for each intrusive memory, to be tallied to arrive at a mean frequency for the memories for the previous day and for the week),” specifically “How often did this memory come up yesterday?” and “How often did this memory come up per day in the past week?” However, this was incorrect, as we had changed this measure at the study start to use the same diary as for the earlier time points (ie, daily diary), that is the “Number of intrusive memories of traumatic event recorded by participants in a diary daily (morning, afternoon, evening, night) per week over the baseline phase and during one week at three month follow-up.” The measure has been updated retrospectively in the CTR for this 3-month period and should be interpreted with caution.

#### Secondary Outcome Measures

*PTSD symptoms* in the past month were evaluated using the PCL-5, a self-report scale with 20 items. Each symptom is rated on a 5-point Likert scale (0=not at all; 4=extremely), with higher scores indicating greater severity. The PCL-5 evaluates the severity of PTSD symptoms according to DSM-5 criteria. It has strong internal and test-retest reliability and good convergent and discriminant validity [27]. Criteria for clinical significance are not available for the PCL-5; however, posttreatment scores of ≤24 can be interpreted as clinically significant change [28]. The Icelandic version of this measure in the SAGA cohort study had excellent internal consistency (*α*=.95).

*Depression symptoms* in the past 2 weeks were evaluated using the Patient Health Questionnaire-9 (PHQ-9), a self-report measure with 9 items rated on a 4-point Likert scale (0=not at all; 3=nearly every day) [29]. The PHQ-9 evaluates depression symptoms and their severity and has good internal reliability and test-retest reliability [29]. The Icelandic version of the SAGA cohort study had good internal consistency (*α*=.89). A 5-point change in the total PHQ-9 score was considered clinically significant [30].

*Anxiety symptoms* in the past 2 weeks were evaluated using the Generalized Anxiety Disorder-7 scale (GAD-7)*.* In this self-report questionnaire, each item is rated on a 4-point Likert scale (0=not at all; 3=nearly every day). The GAD-7 assesses symptoms of general anxiety disorder and its severity and has great internal reliability and good test-retest reliability [31]. In general, the GAD-7 has presumably been useful for screening anxiety disorders [32]. The Icelandic version of the SAGA cohort study had good internal consistency (*α*=.90). A 4-point change on the GAD-7 is considered clinically significant [33].

*Functional impairment* in the previous week was evaluated using the Sheehan Disability Scale (SDS). This self-report measure has three domains: (1) work or school, (2) social, and (3) family life, assessing functional impairment using a 11-point scale (0=not at all; 10=extremely) [34]. A 3-point change on the SDS scale has been considered indicative of response to treatment [35]. To assess the impairment associated with intrusive memories, scale adjustments were made. The SDS has been found to have good internal and test-retest reliability and good construct validity [34]. The Icelandic version of the SDS has been found to have good internal consistency in clinical groups (*α*=.84) [36].

*Self-guided adherence* for daily life using the gameplay part of the intervention was rated “How often did you manage to play Tetris after you experienced an intrusive memory?” (11-point scale: 0=not at all; 10=every time).

*Feasibility and acceptability ratings* for the intervention were assessed with 2 ratings “Would you recommend playing Tetris to a friend?” and “Do you consider gameplay to be an acceptable way to reduce the daily frequency of intrusive memories?” The scores ranged from 0 to 10. Higher scores indicated greater acceptability. Open-ended questions included (1) “How did you feel about playing Tetris after you had an intrusive memory?” and (2) “Did you find the intervention helpful? If yes, how?”

*The impact of intrusive memories on concentration, sleep, and stress* in the past week was evaluated using 6 self-rated items: a total of 2 items assessed general concentration difficulties and difficulties in concentration due to intrusive memories (11-point scales; higher scores indicated greater difficulties), 1 item assessed concentration disruption in minutes in the past week, and 2 items assessed the impact of intrusive memories on sleep (11-point scale; higher scores indicated greater sleep disturbance). An item assessed the impact of intrusive memories on stress levels (0=not at all; 10=affected very much).

*General impact of intrusive memories* was assessed using 2 ratings of intensity and vividness of the intrusions on a 11-point scale (0=not at all; 10=very distressing or vivid).

*Intrusion diary adherence* item was assessed using the rating “How accurately did you fill out the diary?” (0=not at all; 10=very accurate).

*Impact of intrusive memories on daily functioning* was evaluated via an open-ended question: “How have the intrusive memories affected your ability to function in your daily life in the past week?” and a self-rated question, “Have the intrusive memories affected your ability to function in your daily life?” (11-point scale, a higher score indicated greater impact).

### Data Analysis

#### Number of Intrusive Memories

The number of intrusive memories of trauma was recorded by participants in the diary daily (morning, afternoon, evening, and night) during the baseline phase and each week during the intervention phase (weeks 1-6) and during 1 week at the 1-month follow-up. The primary outcome was the change in the number of intrusive memories of the trauma. The timeframe was baseline week to the intervention phase (weeks 1-6) and follow-up (1 month). In practice, owing to scheduling reasons, the baseline phase was longer than 1 week, and as anticipated, the number of intervention weeks varied. Therefore, because these periods had different time lengths, the mean number *per week* was calculated for comparability. Missing data were dealt with by excluding these time points from the calculations and using available data (see *Results* section). For example, a participant had a baseline period of 14 days, but data were present for only 6.5 days; thus, the total number of intrusions per week was calculated as 10 intrusions / 6.5 days × 7 = 10.8 intrusive memories per week during baseline.

When examining change over time, the percentage reduction in total intrusions per week was calculated from the baseline phase to the intervention phase to other periods as follows: 1– (mean number per week during intervention phase / mean number per week during baseline) × 100. For example, for the same participant there were 4.7 intrusions per week in the intervention phase, which was calculated as 1 – (4.7 / 10.8) ×100 = 57% reduction in the intervention phase compared with the baseline.

Please note that at the 3 month-follow-up, we did not use the telephone questions on the CTR (NCT04209283) about the frequency of intrusive memories for the past day or week (eg, “How often did this memory come up per day in the past week?”) but instead replaced this with the same 1 week diary used at earlier time points. We noted the use of a diary at 3 months in our ethics submission, but we incorrectly specified it in our CTR and did not update the CTR on this point until the submission of this paper.

#### Change in the Number of Targeted Intrusive Memories Relative to Nontargeted Memories

We examined the number per week of targeted memories in comparison with nontargeted memories. This was done by calculating the number in the same way as described above for targeted memories (ie, each of targeted memories have different baseline and intervention periods). However, standard baseline and intervention periods were established for nontargeted memories (ie, same length of periods for all nontargeted memories), as they were not targeted by the intervention. The baseline for each nontargeted memory was 1 week (ie, before any memory was targeted), and the intervention phase was determined as the period from when any memory was targeted with the intervention.

#### Other Symptoms and Functioning

A descriptive approach was used to explore whether clinically meaningful changes were observed in the overall symptoms of posttraumatic stress, depression, anxiety, and functional impairment.

### Ethics Approval

This study was approved by the National Bioethics Committee of Iceland (VSNb2017110046/03.01). The participants provided written informed consent before the start of the study. All the sessions followed a written protocol. No adverse events related to the intervention were reported. Participants were asked to briefly consider the visual content of the traumatic memories they selected, which might have resulted in some distress. Previous research has indicated that this intervention approach is well tolerated, including in inpatients with complex PTSD [19]. However, given the early stage of this research, an arrangement was made with an independent clinical psychologist who specializes in trauma for an interview free of charge to the participant and to be referred to a licensed clinical psychologist for treatment if needed. None of the participants had used these services.

### Open Science Statement

The study was registered before the start of the study on ClinicalTrials.gov (NCT04209283) on December 24, 2019. The manuscript contains anonymized summary-level data. The study materials may be made available upon reasonable request with an appropriate material transfer agreement with the University of Iceland or Uppsala. We note that delivery of this intervention at present requires prerequisite training and supervision by psychologists with experience of developing it (see *Procedure*, *Training* section).

## Results

### Overview

The number of intrusive memories and how many were targeted varied among participants. P3 reported having 10 different intrusive memories of physical violence during childhood. In all, 2 of the intrusions were targeted with the intervention; one of the memories (Memory A) was targeted 5 times and the other (Memory B) was targeted once. P3 monitored the memories quite accurately, but data were missing for days 15 to 22 during the intervention phase. P4 reported having 3 intrusive memories of childhood sexual abuse, of which only 1 (Memory A) was targeted with the intervention 5 times. P4 monitored the memories accurately, and data were missing for half of day 1 and for days 8 to 15 during the baseline phase. P5 reported having 6 intrusive memories of childhood sexual abuse, 4 of which were targeted. One of the memories (Memory A) was targeted 3 times, whereas the others (Memories B, C, and D) were targeted once. P5 accurately monitored their memories with no missing data. No attempts were made to retrieve the missing data. On average, 90% of the diary data were completed for all 3 participants. No data were missing for the secondary outcome measures.

### Primary Outcome

#### Change in the Total Number of Intrusive Memories

**Figure 2:**
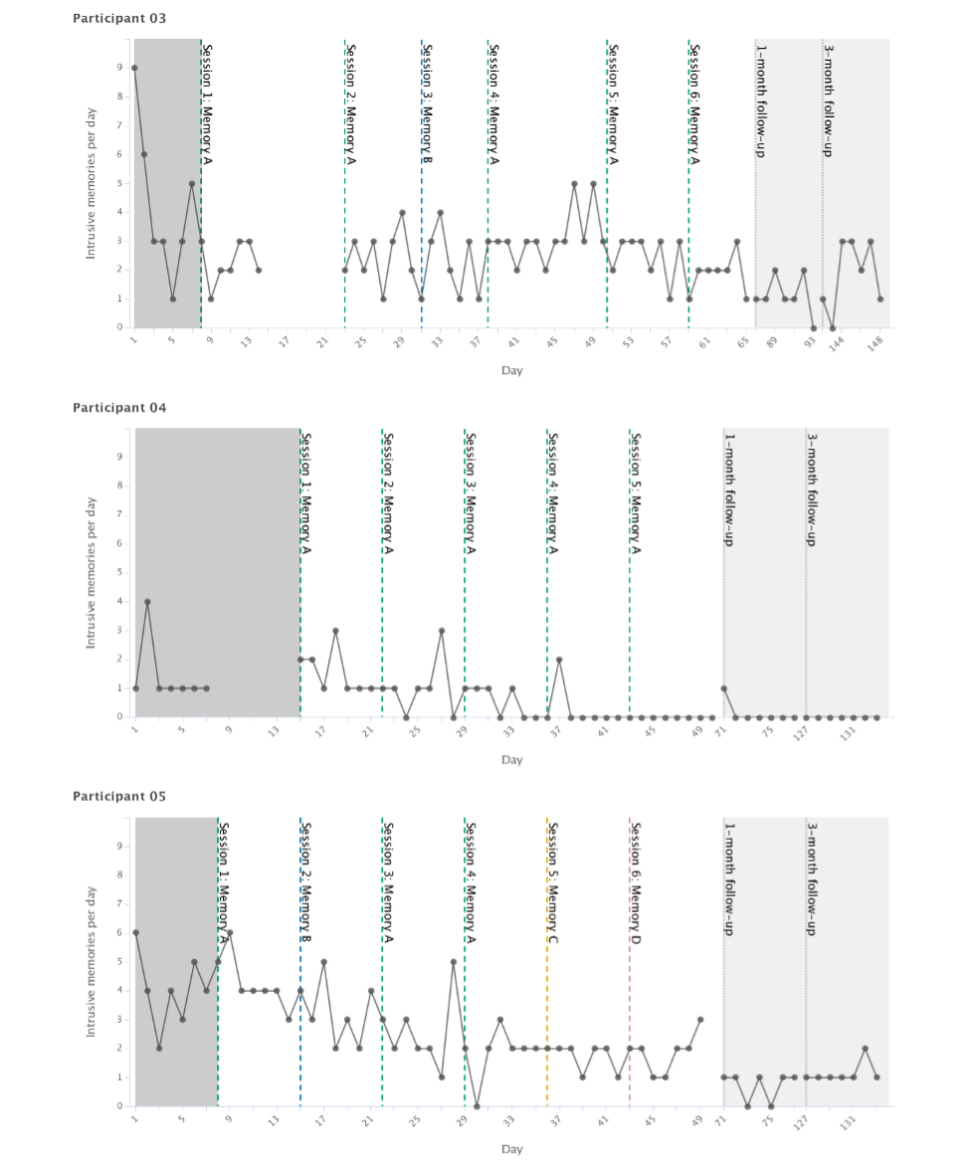
Graphs for visual inspection of primary outcome data for each participant (on the y-axis as total number of intrusive memories per day, ie, for all distinct memories combined). Days since study start are shown on the x-axis, which includes baseline (gray), intervention (white), and follow-up periods (light gray). Dashed vertical lines show when each intervention session was administered and which specific traumatic memory was targeted. Memories are labeled in order of when they were targeted (eg, Memory A was targeted in the first intervention session). Dotted vertical lines show the 1-month and 3-month follow-up periods. Gaps in the time series; for example, in the baseline, represent missing data.

The total number of intrusive memories per day throughout all phases for each participant (baseline, intervention, and 1 month) is shown in Figure 2. In addition, as it is on the same measure, the diary used at 3 months is also shown in Figure 2. Diary compliance was good for the outcome phases, with most missing data in the baseline phase. The number of intrusive memories per day fluctuated during the baseline phase for all the participants (N=3). P3 had 38.8 intrusive memories (summed across all 10 distinct intrusive memories) per week during the baseline phase. The number was reduced to 18.0 per week (54% reduction from baseline) during the intervention phase and further reduced to 8 at the 1-month follow-up week (80% reduction from baseline). P4 had 10.8 intrusions (summed across all 3 memories) per week during the baseline phase, and the number reduced to 4.7 per week (57% reduction from baseline) during the intervention phase and was further reduced to 1 (91% reduction from baseline) at the 1-month follow-up week (Figure 2). P5 had 33.7 intrusive memories (summed across all 6 distinct intrusive memories) per week during the baseline phase, which reduced to 20.7 per week (39% reduction from baseline) during the intervention phase. The number further reduced at the 1-month follow-up to 5 that week (85% reduction from baseline).

We now consider each of the participant graphs shown in Figure 2. Visual inspection of P3 showed that relative to baseline after the first intervention session, the number of intrusions decreased. The number persisted with some fluctuation through the intervention period (days 8-65) and the 1-month and 3-month follow-ups. Visual inspection for P4 showed that relative to baseline (which included missing data), the number of intrusions remained relatively steady until the fourth intervention session targeting the same memory (day 36), when there was a noticeable drop in occurrence to 0 that was maintained in the last intervention session and 1-month (and 3-month) follow-up. Visual inspection of P5 showed a slight drop in the frequency of intrusions after the second intervention session with some fluctuations, until intervention session 5 (day 36), where the reduction in frequency became more stable (Figure 2). The frequency decreased even further at the 1-month follow-up (and at 3 months).

#### Data for the 3-Month Follow-up Diary

P3 had 13 intrusive memories in their diary at the 3-month follow-up (67% reduction from baseline). P4 had 0 intrusive memories (100% reduction from baseline) at the 3-month follow-up, whereas P5 had 8 in the week of the 3-month follow-up (76% reduction from baseline; Figure 2). Patterns in relation to diaries at earlier time points are noted in the *Change in the Total Number of Intrusive Memories* section; however, please see earlier notes about the change in measures at this time point in contrast to the original CTR.

#### Change in the Number of Targeted Intrusive Memories Relative to Nontargeted Memories

The number of targeted and nontargeted intrusions per week at baseline, in the postintervention phase, and at the 1- and 3-month follow-ups are displayed in Table 1. The mean number of individual targeted memories was 7.6 (SD 4.3) per week in the baseline phase and reduced to 5.8 (SD 2.7) per week in the intervention phase. For individual memories, refer to Table 1. However, for nontargeted intrusions, the baseline rate for individual memories was very low, such as 1 per week. Therefore, these percentages are not included in Table 1. The mean number of nontargeted memories was 2.5 (SD 3.3) per week in the baseline phase and reduced to 0.2 (SD 0.4) memories per week in the intervention phase. The number of targeted memories continued to decrease at the 1-month follow-up week to 2.0 (SD 2.5), and nontargeted memories were reduced to 0 memories. At the 3-month follow-up, the mean number of targeted memories was 3.0 (SD 3.4), and the frequency for nontargeted memories was again 0 that week.

**Table 1:** Number of intrusive memories per week in the baseline phase, postintervention phase, and at 1 and 3-month follow-ups from baseline phase for each participant (P)^a^.

Participant and intrusions		Baseline phase	Postintervention phase	1-month follow-up	3-month follow-up	
**P3**						
	Memory 1 (A^b^)	14	11.2	6		3
	Memory 2 (B^b^)	8.8	6.4	2		10
	Memory 3 (C)	6	0.3	0		0
	Memory 4 (D)	1	0	0		0
	Memory 5 (E)	0	0	0		0
	Memory 6 (F)	3	0	0		0
	Memory 7 (G)	1	0	0		0
	Memory 8 (H)	3	0.1	0		0
	Memory 9 (I)	0	0	0		0
	Memory 10 (J)	2	0	0		0
	Total	38.8	18.0	8		13
	Total targeted^b^	22.8	17.6	8		13
	Total nontargeted	16	0.4	0		0
**P4**						
	Memory 1 (A)^b^	8.6	3.9	1		0
	Memory 2 (B)	1.1	0.8	0		0
	Memory 3 (C)	1.1	0	0		0
	Total	10.8	4.7	1		0
	Total targeted^b^	8.6	3.9	1		0
	Total nontargeted	2.2	0.8	0		0
**P5**						
	Memory 1 (A)^b^	10.6	6.5	0		2
	Memory 2 (B)^b^	7	2.8	0		2
	Memory 3 (C)^b^	2.6	4.2	0		4
	Memory 4 (D)^b^	2.0	5.6	5		0
	Memory 5 (E)	11.6	0.5	0		0
	Memory 6 (F)	0	1	0		0
	Total	33.7	20.7	5		8
	Total targeted^b^	22.1	19.2	5		8
	Total nontargeted	11.6	1.5	0		0

^a^Total intrusions do not equate to the sum of intrusions for each memory here because the length of the baseline and intervention phases differ across each memory and the total. See the *Data analysis* section for further details.

^b^Specific intrusive memories targeted by the intervention.

### Secondary Outcomes

#### Ratings of Adherence to Completing the Diary, General Impact of Intrusive Memories (Vividness and Distress), and Use of the Intervention in Daily Life

Self-reported accuracy for filling out the daily intrusion diaries was high throughout the study period (Table 2). For the general impact of intrusive memories, the ratings for vividness of the intrusions did not change considerably, although if anything, showed some decline (Table 2). Distress associated with intrusions more clearly diminished during the intervention and follow-up phases for all 3 participants (Table 2). For use of the gameplay intervention in daily life, ratings of the self-guided adherence to Tetris gameplay after experiencing an intrusive memory between sessions are also shown in Table 2.

**Table 2:** Ratings for adherence to intrusive memory diary, general impact of intrusive memories, and use of the gameplay intervention in daily life (N=3).

Ratings and participant			Session 1		Session 2		Session 3		Session 4		Session 5		Session 6		1-month follow-up		3-month follow-up
**Diary accuracy^a^**																	
	P^b^3	8		7		8		7		8		7		7		7	
	P4	9		8		7		9		8		—^c^		8		10	
	P5	8		9		8		8		8		8		7		9	
**Intrusions’ vividness^d^**																	
	P3	8		8		7		8		7		6		6		7	
	P4	7		6		5		7		4		—		5		0	
	P5	7		8		7		7		7		6		6		5	
**Intrusions’ distress^e^**																	
	P3	7		9		7		7		7		6		6		4	
	P4	6		5		4		4		2		—		1		0	
	P5	8		8		7		7		7		6		5		3	
**Intervention use in daily life^f^**																	
	P3	—		3		3		1		4		3		1		1	
	P4	—		3		2		7		0		—		0		0	
	P5	—		7		7		6		8		8		6		2	

^a^How accurately did you complete the diary? 0=not at all; 10=very accurately.

^b^P: participant.

^c^Missing data.

^d^During the last week, how vivid was your intrusive memory? 0=not at all; 10=very vivid.

^e^During the last week, how distressing were your intrusive memories? 0=not at all; 10=very distressing.

^f^How often did you manage to play Tetris after you experienced an intrusive memory? 0=never; 10=every time.

#### Feasibility and Acceptability of Using a Computer Gameplay Intervention

Participants were asked if they would recommend the intervention to a friend. P3 rated this item at 9, P5 gave it a rating of 8 (highly likely to recommend to a friend), and P4 rated the item at 3 (unlikely to recommend to a friend). They also rated if they considered gameplay to be an acceptable way to reduce intrusive memories. P3 and P5 rated the item at 8 (high acceptability), and P4 rated it at 3 (low acceptability). When participants were asked to rate how they felt about playing Tetris after having an intrusion, P3 noted that “It reduced the emotion, sometimes I was able to concentrate and think my way through it. Sometimes I experienced a kind of peace within.*”* P4 said, “I played so there was no room for other thoughts,” and P5 reported, “Sometimes it’s difficult to play for 25 minutes, but I played as many times as I could, for 5 to 25 minutes.” When the participants were asked if they found the intervention helpful, P3 reported, “When I was able to plan ahead while playing the game, my brain could not interrupt me. The emotion that causes distress fades.” P4 said, “Yes, I could not think about anything else whilst playing,” and P5 reported, “I felt a physical calmness, like the pit in my stomach was shrinking.”

#### Self-report Measures for Posttraumatic Stress, Depression and Anxiety Symptoms, and General Functioning

There was a clear reduction in posttraumatic stress symptoms (PCL-5) from baseline to the postintervention phase, which tended to continue to drop further during follow-up for all 3 participants who completed the intervention, suggesting clinical improvement (Table 3). Depression symptoms (on the PHQ-9) and anxiety symptoms (on the GAD-7) seemed to follow a similar pattern for 2 (P3 and P5) out of the 3 participants, showing reductions in the postintervention phase and during follow-up. Functional impairment (as measured by the SDS) was reduced for the same 2 out of 3 participants (P3 and P5) from baseline to the postintervention phase and at the 1-month follow-up, but for P3, it increased at 3 months. It should be noted that P4’s ratings for all of these measures were low at baseline (Table 3).

**Table 3:** Self-report measures of secondary outcomes (posttraumatic stress, depression and anxiety symptoms, and general functioning) and impact of intrusive memories on concentration, sleep, stress, and daily functioning for each participant (P).

Item and participant			Baseline interview		Postintervention interview		1-month follow-up		3-month follow-up
**PCL-5^a^**									
	P1	6		—^b^		—		—	
	P2	54		—		—		—	
	P3	54		35		20		35	
	P4	26		14		6		11	
	P5	64		49		51		31	
**PHQ-9^c^**									
	P1	3		—		—		—	
	P2	19		—		—		—	
	P3	13		8		6		9	
	P4	5		3		3		2	
	P5	19		11		14		11	
**GAD-7^d^**									
	P1	2		—		—		—	
	P2	8		—		—		—	
	P3	14		7		6		10	
	P4	2		3		5		1	
	P5	18		9		17		4	
**SDS^e^**									
	P1	7		—		—		—	
	P2	20		—		—		—	
	P3	20		16		13		21	
	P4	4		3		0		0	
	P5	21		16		8		7	
**Concentration disruption related to intrusive memories^f^**									
	P1	3		—		—		—	
	P2	3		—		—		—	
	P3	7		5		3		5	
	P4	2		2		1		0	
	P5	7		4		4		3	
**General concentration^g^**									
	P1	0		—		—		—	
	P2	6		—		—		—	
	P3	7		6		5		6	
	P4	2		2		2		0	
	P5	7		4		3		—	
**Duration of disruption^h^**									
	P1	1		—		—		—	
	P2	3		—		—		—	
	P3	4		4		3		4	
	P4	2		1		2		1	
	P5	4		4		2		2	
**Sleep^i^**									
	P1	0		—		—		—	
	P2	5		—		—		—	
	P3	4		4		3		4	
	P4	1		1		1		0	
	P5	10		8		7		5	
**Nightmares^j^**									
	P1	0		—		—		—	
	P2	4		—		—		—	
	P3	7		2		2		4	
	P4	0		1		1		0	
	P5	10		8		8		5	
**Stress^k^**									
	P1	0		—		—		—	
	P2	4		—		—		—	
	P3	6		2		0		2	
	P4	1		1		2		0	
	P5	7		3		3		3	
**Daily functioning^l^**									
	P1	0		—		—		—	
	P2	8		—		—		—	
	P3	7		5		2		5	
	P4	0		0		0		0	
	P5	7		0		2		0	

^a^PCL-5: Posttraumatic Stress Disorder Checklist-5; score range 0 to 80.

^b^Missing data.

^c^PHQ-9: Patient Health Questionnaire-9; score range 0 to 27.

^d^GAD-7: Generalized Anxiety Disorder-7 scale; score range 0 to 21.

^e^SDS: Sheehan Disability Scale; score range 0 (unimpaired) to 30 (highly impaired).

^f^In the past week, how much did your intrusive memories disrupt your concentration? 0=not at all disruptive; 10=extremely disruptive.

^g^In the past week, how much difficulty did you have concentrating generally? 0=no concentration difficulty at all; 10=extreme concentration difficulty.

^h^When you had an intrusive memory, how long did it disrupt your concentration (in minutes) in the past week? 0 (<1 min) to 5 (>60 min).

^i^Did your intrusive memories interfere with sleep during the night in the past week? 0=not at all; 10=interfered very much.

^j^Did you experience any nightmares that interfered with your sleep during the night in the past week? 0=did not experience any nightmares; 10=experienced a lot of nightmares.

^k^In the past week, did your intrusive memories affect how stressed you felt? 0=not at all; 10=affected very much.

^l^Have the intrusive memories affected your ability to function in your daily life? 0=not at all; 10=affected very much.

#### Impact of Intrusive Memories on Concentration, Sleep, Stress, and Daily Functioning

The ratings of the impact of intrusive memories on concentration, stress, and sleep are included in Table 3. P5 reported improved concentration after her intrusions over the course of the intervention and follow-up period. At baseline, when P5 experienced an intrusive memory, her concentration was disrupted for 10 to 30 minutes on average, and this time was reduced to 1 to 5 minutes at follow-up. P3 reported a slight improvement in concentration with respect to intrusive memories specifically but not in general. Ratings on concentration disruption from intrusive memories were low at baseline for P4 and did not change in the postintervention phase. The effect of intrusive memories on sleep did not change for P3 or P4, whereas P5 showed some reduction. Nightmares were reduced for both P3 and P5, whereas nightmares were almost nonexistent for P4. The stress levels associated with intrusions were reduced during the intervention and follow-up periods for P3 and P5.

The reported impact of intrusive memories on the ability to function in daily life over the intervention and follow-up periods showed improvement (except for P4, who scored 0 at baseline). At baseline, the participants were asked to respond to an open question about how their intrusive memories impacted their ability to function in daily life. At baseline, P3 said, “When I have this overwhelming feeling, I find it difficult to be around other people, the worst thing is how it affects my ability to stay present for my daughters.” P4 said, “I have anger inside me, I constantly think back to how no-one noticed what was done to me.” P5 reported, “I get very stressed and anxious; it takes a lot of energy to get out of the emotion...” In the last intervention session, P3 reported, “The intrusions disrupt my concentration,*”* and P5 said, “I experience distress and nightmares.” At the 1-month follow-up, P3 disclosed, “When I am under a lot of stress, they disturb me more.” P5 reported, “The images no longer have color, cause less disruption and are less frequent.” At the 3-month follow-up, P3 said, “I am under a lot of stress and dealing with a certain communication problem which triggers my PTSD and the intrusions” and that “I can now comprehend that this is only a memory, and I don’t feel as distressed.” P5 reported, “They mostly impact my anxiety” and “I am not a person that easily believes in things, but this intervention works.” For all phases following the baseline, P4 reported, “The memories have no impact anymore.”

## Discussion

### Principal Findings

The aim of this case series was to extend our previous case study by evaluating a novel visuospatial intervention designed to reduce the number of intrusive memories of trauma [6,14,37]. The intervention was adapted to the Icelandic setting from previous clinical studies [6,19,20]. The total number of intrusive memories per week (primary outcome) was reduced between 38% and 56% from baseline to the intervention phase, in line with earlier results found by Kessler et al [19] and what we found in our earlier case study [6]. Importantly, we also found in this case series that the frequency of intrusive memories continued to decrease at the 1-month follow-up (the reduction from baseline was approximately 85%). In the diary used at the 3-month follow-up, the reduction from baseline was from 66% to 100%; although this measure replaced another one and was not prespecified, further investigation is required. These results are similar with what we found in the earlier case study [6] in which the frequency also continued to reduce from 52% in the postintervention phase to 76% at the 1-month follow-up and to 92% at the 3-month follow-up. These results indicate that the reduction in the frequency of intrusions might continue rather than rebound, which could be because of the simplicity of self-administered use of the intervention, giving participants the chance to use it independently, if needed. Reductions in distress related to intrusive memories were evident in all participants. A limitation of the study is that the 3-month diary data must be treated as exploratory, as it was not preregistered in the CTR (though it was in our ethics approval), and further studies should include this.

Not only did the targeted intrusions reduce in this case series but nontargeted intrusions were also reduced in the intervention phase. Although this appears relatively more so than the targeted ones, results must be treated with caution because of the potential floor effects on the low baseline number of nontargeted intrusions rendering comparisons misleading. Kessler et al [19] found that overall, targeted memories were reduced by 64% and nontargeted memories by 11%. The sample of participants in the study by Kessler et al [19] was inpatients with a diagnosis of complex PTSD with a larger number of different memories and baseline symptom rates, whereas the participants in this case series were non–treatment seeking with less symptom severity. Furthermore, targeted memories were reported to be much more distressing than nontargeted ones and may, in some cases, need more time to reduce in frequency (hence, the long-term effect described earlier in this section). It would be clinically interesting in future studies to see if there were links between treating a memory (say from the same trauma) that could generalize to reductions in nontargeted memories of the same episode.

We examined whether a reduction in the number of intrusions would have an impact on the symptoms of posttraumatic stress, depression and anxiety symptoms, and general functioning (secondary outcomes). The general pattern was that posttraumatic stress symptoms were reduced for all participants in the postintervention phase (cutoffs for clinical significance were not available for this measure). Depression and anxiety symptoms were reduced at times suggestive of a clinically significant change for 2 (P3 and P5) of the 3 participants in the postintervention phase [30,33], whereby a 5-point change in the PHQ-9 total score and a 4-point change on the GAD-7 can be considered clinically significant. Symptoms tended to be reduced further at follow-up. It should be noted that P4 had very low levels of distress, depression and anxiety symptoms, and impaired function at baseline. The overall findings are similar to those of our previous case study [6] and the study by Kessler et al [19]. These results provide preliminary evidence that a reduction in the number of intrusions from using this intervention could possibly reduce other symptoms connected to intrusive memories after trauma and improve functioning, with important implications for the quality of life. For the 2 (out of 3) participants who had a reduction in posttraumatic stress symptoms, depression and anxiety, and impairments in concentration and other factors related to intrusive memories, the overall pattern for secondary measures was that some improvements tended to continue for a longer term.

P3 and P5 rated the intervention as acceptable; using Tetris gameplay was an acceptable method to reduce the frequency of their intrusive memories and noted that they would recommend the intervention to a friend. This is similar to what Holmes et al [38] found among refugees and what we found in our earlier case study [6]. However, P4 did not rate the intervention as acceptable and was unlikely to recommend it to a friend, although she noted that it was helpful. It is important to further develop how the intervention can be made more feasible and acceptable to a range of users.

Two of the participants ceased participation in the study after the baseline phase; the reasons were unrelated to the intervention but were related to scheduling issues in their daily life. However, it is important to determine who is most likely to benefit from the intervention and how to educate individuals about it in a way that increases the chance of people making an informed choice of whether they are able to try it or have the time to take part in a course of treatment. It could also be explored whether the intervention could have an impact on the frequency of intrusive memories with fewer guided intervention sessions, which would reduce the participation load.

Owing to the COVID-19 pandemic, the delivery of the intervention was changed from face to face to remote (eg, web-based communication via Kara Connect). Originally, our plan was to gradually move toward remote delivery in future studies. When the pandemic struck, it forced the change to occur more quickly during the intervention phase of the study. This turned out to be a positive development, as the data did not suggest that the intervention became less effective by being delivered remotely. Recent research by Singh et al [39] indicated that this novel intervention delivered remotely could be an acceptable method to reduce the number of intrusive memories among health care staff. The number of intrusive memories was reduced to 0 at the 5-week follow-up in all 3 participants [39]. Continued remote delivery using a web-based platform instead of face-to-face delivery will be important in future studies [40], thereby removing geographical restraints and making it possible to reach people regardless of where they live or whether they are in quarantine (eg, owing to the COVID-19 pandemic) [22].

### Conclusions

Targeting established intrusive memories of trauma that participants had been experiencing for some years (eg, from childhood sexual abuse) with a brief visuospatial intervention, involving a brief memory reminder and Tetris gameplay with mental rotation, seems to show promise for further exploration as a method to reduce their frequency. These early data suggest that the intervention might also result in symptom reduction related to posttraumatic stress, anxiety and depression, and improved functioning; however, further studies are needed. Because of its simplicity, this intervention might be capable of removing common barriers to existing treatment options after trauma, such as for PTSD, including some patients’ reluctance to talk about and describe their trauma in detail to a therapist, high costs, and a limited number of qualified therapists [9]. The intervention might even be delivered by nonexperts in evidence-based trauma-focused therapy after brief training, with ongoing supervision—something that should be further examined.

The results of this study are encouraging, and the effects of the intervention on the number of intrusive memories need to be further explored. Continuing to develop this kind of scalable intervention is crucial to reach a large number of people in need of treatment after experiencing trauma. Future research should further examine the feasibility and acceptability of remote delivery by nonexperts in mental health (rather than only qualified clinical psychologists) and whether fewer intervention sessions can yield similar results. Randomized controlled trials are required to assess the intervention.

## Abbreviations

CAPS-5: Clinician-Administered Posttraumatic Stress Disorder Checklist-5 Scale for Diagnostic and Statistical Manual of Mental Disorders, Fifth Edition
CTR: clinical trial registration
DSM-5: Diagnostic and Statistical Manual of Mental Disorders, Fifth Edition
GAD-7: Generalized Anxiety Disorder-7 scale
MINI: Mini International Neuropsychiatric Interview
PCL-5: Posttraumatic Stress Disorder Checklist-5
PHQ-9: Patient Health Questionnaire-9
PTSD: posttraumatic stress disorder
REDCap: Research Electronic Data Capture
SAGA: Stress-And-Gene-Analysis
SDS: Sheehan Disability Scale
